# Analysis of the Diet Quality and Nutritional State of Children, Youth and Young Adults with an Intellectual Disability: A Multiple Case Study. Preliminary Polish Results

**DOI:** 10.3390/nu13093058

**Published:** 2021-08-31

**Authors:** Michał Skrzypek, Wojciech Koch, Karolina Goral, Klaudia Soczyńska, Olga Poźniak, Klaudia Cichoń, Olga Przybysz, Marcin Czop

**Affiliations:** 1 Department of Clinical Dietetics, Medical University of Lublin, Chodźki 1 Street, 20-093 Lublin, Poland; karolina.goral@umlub.pl (K.G.); klarysa1196@gmail.com (K.S.); olga.poz@wp.pl (O.P.); klaudiakrysa@wp.pl (K.C.); olgaprzybysz2506@gmail.com (O.P.); 2 Department of Food and Nutrition, Medical University of Lublin, Chodźki 4a Street, 20-093 Lublin, Poland; 3 Department of Clinical Genetics, Medical University of Lublin, Radziwiłłowska 11 Street, 20-080 Lublin, Poland; marcin.czop@umlub.pl

**Keywords:** neurodevelopmental disorders, intellectual disability, Down Syndrome, nutritional status, dietary intake

## Abstract

(1) Background: Considering the limited amount of available data on the diet quality of children, adolescents, and young adults with an intellectual disability (ID) in Poland, as well as the higher incidence of nutritional disorders among people with ID in comparison to the general population, a study was conducted to assess the quality of diet in community-dwelling young individuals with ID. (2) Methods: A multiple-case study was carried out to obtain data on anthropometric parameters and food records over a three-day observation period for 18 subjects with ID. The nutritional value of the products and dishes consumed by the subjects was calculated using the commercial computer program Kcalmar.pro (Hermax, Poland), which contains the Polish database of the nutritional value of food products and dishes. The results presented here are those of a pilot study of a wider research project. (3) Results: The study group consisted of seven women (38.9%) and 11 men (61.1%) with an average age of 12.94 years (range 4.00–22.00) and an average BMI of 25.2 (range 14.5–35.4). The obtained results confirmed the suboptimal pattern of nutrition in children and adolescents with ID. Regarding energy intake and dietary macronutrients, only in 27.8% of cases, energy intake exceeded the Estimated Energy Requirement corresponding to age, sex, body weight, and height. Most of the respondents were characterized by correct intake of proteins, fats, and carbohydrates, and 83.3% showed excessive intake of saturated fatty acids. Excessive intake of vitamins B1, B2, and B6 was observed in all subjects, and that of vitamins B3 (niacin), B12, A, and C in the vast majority of subjects, while deficiency of vitamin D was observed in all individuals and folic acid in 22.2% of individuals. Excessive intake of minerals, such as sodium, phosphorus, and magnesium was noted among most of the respondents, while the intake of calcium and iodine was found to be insufficient. Compared to people with less severe ID (grades 1 and 2), people with grade 3 ID showed significantly lower intake/reference fulfillment of dietary components such as vitamin B6, potassium, phosphorus, iron, copper, iodine, magnesium, and zinc. No significant differences were noted in the nutritional reference fulfillment for various nutrients among the groups distinguished by sex, nutritional status, or the cause of ID. (4) Conclusion: Health supervision guidelines proposed for persons with ID should take into account the dietary practices of the families caring for them, with an emphasis on the prevention and correction of irregularities that may reduce the effectiveness of rehabilitation and deteriorate the health of the affected people. Caregivers/parents of each person with ID who took part in the study were given individualized dietary advice aimed at correcting the dietary abnormalities.

## 1. Introduction

People with intellectual disability (ID) are characterized by a high prevalence of abnormal eating patterns, as well as a high risk of nutritional disorders [1,2,3,4,5,6], especially overweight and obesity [7,8,9,10]. In the general population, ID occurs at an average frequency of 1–3% and is caused by the interaction of many diverse genetic and environmental factors. Genetic factors account for 17–50% of the ID cases [11], and the most common chromosomal aberration that causes ID is Down syndrome (DS). The incidence of DS is approximately 1/800 births worldwide [12] and varies among different countries depending on the willingness of societies and parents to deliver neonates with DS diagnosed in prenatal testing. It is estimated that there are about 60,000 people living with DS in Poland [13]. In the population of children with DS aged 6 months to 6.5 years, the prevalence of disabilities related to food and feeding is determined as 80% [14].

The widespread prevalence of eating disorders in the population with various types of ID is predominantly due to the limited understanding of the principles of rational nutrition, and the dependence of this group on caregivers in terms of food choices. Depending on the principal clinical diagnosis, individuals with ID may have structural and/or functional gastrointestinal tract disorders affecting dietary intake and nutrient absorption. In addition, the individuals may experience disturbances in appetite, food preferences, and taste perception which will also affect their dietary intake. The energy balance in people with ID is influenced by the lower level of physical activity, resulting, among others, from disorders of the body structure, including defects in the skeletal and muscular systems. The nutritional status of this group is also more commonly affected by metabolic and hormonal disorders, in comparison to the general population [1,2,3,4,5,6,7,8,9,10]. A factor increasing the risk of excess body weight in people with ID may also be the greater dimension of “screen time”—understood as the overall amount of time spent sitting while using electronic devices with a screen—resulting from social isolation [10]. Dietary behavioral problems observed among the ID population also include food neophobia and food fixations that hinder the changes in the eating habits. Such a situation may result from a psychological need to stay safe [15]. Furthermore, pharmacotherapy can promote abnormal eating patterns in people with ID. Nutritional deficiencies in individuals with ID lead to the development of comorbidities and further deterioration of their health [16].

Moreover, the chewing and swallowing difficulties, as well as delayed eruption of deciduous teeth, in children with DS which delay the introduction of solid foods additionally may cause nutritional deficiencies [16]. Children with DS prefer simple carbohydrates and foods that are easy to chew and swallow, which results in the rejection of fresh fruits and vegetables from their diet. Moreover, nutritional deficiencies in DS are associated with faster progression of degenerative changes in the central nervous system, including those related to early-onset Alzheimer’s disease [16].

The current literature has no studies from Poland on the diet quality of children, adolescents, and young adults with ID, with reference to the nutritional intake of individual dietary components. There are only few studies available on this topic in world literature, which were discussed in the Discussion section.

The aim of this study was to evaluate the nutritional status and intake of various nutrients with daily food rations in children, adolescents, and young adults with ID. The choice of ID as a criterion for inclusion in research is motivated by the fact that ID is the endpoint of a series of specific clinical problems, which generates severe effects for patients and their families with respect to the demand for care, including nutritional care, and limitations in social functioning. Furthermore, ID is a criterion for participation in the system of social support dedicated to persons affected by different clinical problems resulting in the impairment of intellectual abilities. Although the study group was small and quite diverse in terms of age, the obtained results are the first of this type in Poland and may constitute an important starting point for further research in the future. However, it should be remembered that access to such patients, as well as the methodology of the conducted research, are very difficult, which significantly reduces the size of the studied population. The results reported here are those of a pilot study of a wider research project aimed at assessing the quality of diet in a representative sample of individuals with ID selected from the Polish population.

## 2. Materials and Methods

### 2.1. Sample and Procedure

Data for the study were obtained from 18 community-dwelling individuals with ID (7 females, 11 males; mean age 12.9, minimum 4 years, maximum 22 years, M 13, SD 5.8; people diagnosed with DS were included in the ID category due to the constant presence of mental retardation of various degrees in the clinical picture of DS). The respondents were recruited from the following institutions, which are dedicated to the care and support of people with ID and their families (disability services): Daily Activity Center of the Polish Association for People with Intellectual Disability; Circle in Lublin, at the seat of the Association for People with Intellectual Disability “Camino” in Lublin and the Association of Families of People with Down Syndrome “Hidden Treasure” in Lublin. A total of 18 parents/guardians of children with ID were invited to participate in the study. All 18 agreed, and their children were included in the study. Parents/guardians provided data on the diet quality of participants with ID. All participants of the study received an individual diagnosis of the nutritional status and assessment of diet quality as well as dietary advice aimed at the correction of any irregularities found in this regard, along with a proposal of a 7-day menu.

Participation in the study was voluntary and anonymous. The research sample was selected based on purposeful selection. The exclusion criterion for the study was the presence of diseases causing significant nutritional impairment or diseases requiring the use of special diets.

Socio-economic situation was evaluated based on the KomPan questionnaire developed and validated by the Polish Academy of Sciences [17]. Parents/guardians of the respondents indicated a city of over 100,000 inhabitants as their place of permanent residence (n = 15, 83.3%) or rural areas (n = 3, 16.7%). Financial situation was rated as below average by 5.6% of parents/guardians (n = 1), average by 72.2% (n = 13), and above average by 22.2% (n = 4). The distribution of the assessments of financial situation by the parents/guardians was as follows: we live modestly—we must be very economical on a daily basis: 5.5% (n = 1); we live on average—it is enough for us every day, but we have to save for more serious purchases: 39% (n = 7); we live well—it is enough for us without special saving: 50% (n = 9); and we live very well—we can afford some luxury: 5.5% (n = 1).

The study was conducted from 28 February 2019 to 26 September 2019.

### 2.2. Measurements

Trained staff obtained measurements of subjects by following standardized techniques. Nutritional status of the subjects was evaluated using anthropometric measurements, taking into account their body weight, height, and BMI (calculated as body weight divided by the square of height, in kg/m^2^). The anthropometric measurements were obtained according to the World Health Organization (WHO) standards [18], considering body weight to the nearest 0.1 kg and height to the nearest 0.1 cm, using TANITA HR-001 mobile height measuring device and TANITA BC-587 scale (TANITA, Tokyo, Japan). This assessment was conducted in a specific room in the above-enumerated institutions, in the presence of the parents/guardians of the children with ID.

To assess the nutritional status, the calculated BMI value was compared to the current Polish norm expressed in the form of a percentile grid, dedicated to the children of both sexes aged 3–18.

In addition, due to the lack of a Polish standard dedicated to children with DS, the US percentile grids of the BMI index were used to evaluate the nutritional status of DS patients aged 2–20 years [19]. The percentile band determined by the percentile lines from 75 to below the 95th percentile was adopted as the overweight range, while for obesity the percentile band at the level of the 95th percentile or above was adopted. The application of norms specific for people with DS is necessary, due to the shorter length of limbs in people with DS compared to those without DS, which results in an altered distribution of body weight in relation to height [19].

To assess the diet/nutrition quality, the parents/guardians of the respondents with ID involved in the study were asked to complete a 3-day dietary record questionnaire (2 working days—school—and one weekend/free from school). The days on which the nutritional pattern was recorded were selected by parents/guardians of the subjects with ID. All participants in the study were asked to select the days in which the nutritional patterns of a person with ID were typical of/usual for this person. During the first meeting with a dietitian, parents/guardians of the subjects were given precise instructions concerning the completing of the dietary record questionnaire. Parents/guardians of the subjects were asked to bring the filled questionnaire to the second meeting with the team of dietitians, during which the dietary record was verified with regard to correct completion. Incomplete quantitative and/or qualitative data on the meals consumed by the subjects, during the three days of conducting the dietary record, were then completed by a dietitian based on a face-to-face interviews with parents/guardians of the subjects. During the second meeting with the team of dietitians, anthropometric measurements of the subjects were also carried out. Because of the burden of the parents/guardians of the subjects with ID, only a single dietary record was possible. In general, 3-days dietary survey was used in cooperation with dietitian helping parents/guardians to complete dietary questionnaires. The limitation of the performed method is that the days were arbitrarily chosen by parents/guardians.

The adopted qualitative and quantitative methods of diet evaluation provide information about the composition of meals, and frequency and amount of food products or nutrients consumed. In combination with a computer program, these methods allow assessing the intake of dietary components. To clarify the portion size of products and meals consumed, an album containing photos of products and dishes, which is an appropriate tool of such a type officially used in Poland, was used [20]. The reported size of portions was verified by certified dietitians through face-to-face interviews. The nutritional value of the consumed products and dishes was calculated using the commercial computer program Kcalmar.pro (Hermax, Lublin, Poland), which is equipped with the officially used Polish database of the nutritional value of food products and dishes [21]. Dietary records made by the guardians/parents of the subjects with ID were entered into the program Kcalmar.pro by certified dietitians (KS, OP, KC and OP) and subsequently verified by a clinical dietitian/Medical University of Lublin researcher (KG) with the longest professional and work experience. The dietary records obtained from the guardians/parents of the subjects with ID are available on demand.

Based on the 3-day dietary records filled in by the parents/guardians of persons with ID, the energy value of daily food rations (DFR) and levels of nutrient intake (content of nutrients in the DFR) were calculated and compared to the nutrition standards for the Polish population, appropriate for a given age group. The obtained results were also compared with the national consumption standards at the level of Estimated Average Requirement (EAR) for individual dietary components (if available), applying the age-appropriate standard for a specific examined person [22,23,24]. In the case of dietary ingredients for which the norm was not defined at the EAR level, the norm was referred to the level of Recommended Dietary Intake (RDA) or Adequate Intake (AI).

For assessing consumption and interpreting the implementation of the norm, the so-called conservative approach, which involves the analysis of difference between the observed consumption/intake and the level of the EAR norm (average group requirement) for age and sex, was used [25]. It was arbitrarily assumed that the permissible deviation from the norm should not exceed 10%. In a situation where a given person consumed at a level within the range of 90–110% of the EAR standard, the consumption was assumed as normal; when the consumption was below 90%, it was assumed as deficient; and when the consumption was above 110%, it was assumed as excessive. When AI was used as the reference, a different interpretation was made: consumption at or above the AI level was considered sufficient, while consumption below the AI level was considered insufficient.

The study evaluated the intake of the following nutrients based on appropriate references [23,25]:

- Total proteins (g)
- Total fats (g)
- Saturated fatty acids (SFA) (g)
- Total carbohydrates (g)
- For the examined persons with abnormal body mass, the macronutrient intake with the diet was additionally referred to the Total Energy Requirement (TEE), calculated for appropriate body mass. For the subjects for whom percentile grids were applied to estimate their nutritional status, appropriate body mass was calculated considering the BMI for the 50th percentile for age, sex and height of the examined person. In the case of subjects with DS with improper body mass, for whom, because of their age, it was impossible to use percentile grids for children and adolescents with DS, appropriate body mass was assessed using the transformed BMI formula, based on the height of the subjects. The BMI value of 24.9 kg/m^2^ was adopted as normal. The norm for protein, fat and carbohydrates was expressed as the gram equivalent of 10–20%, 20–35% and 45–65% of TEE respectively.
- Dietary fiber (g) (AI)
- Sodium (g) (AI)
- Calcium (mg) (EAR)
- Iron (mg) (EAR)
- Magnesium (mg) (EAR)
- Zinc (mg) (EAR)
- Copper (mg) (EAR)
- Iodine [μg] (EAR)
- Manganese (mg) (AI)
- Vitamin D (μg)(AI)
- Folates (μg)(EAR)
- Vitamin C (mg) (EAR)
- Vitamin E (mg) (AI)
- Thiamine (mg) (EAR)
- Riboflavin (mg) (EAR)
- Niacin (mg) (EAR)
- Vitamin B6 (mg) (EAR)
- Vitamin B12 (mg) (EAR)
- Vitamin A (μg)(EAR)

When calculating the total daily energy supply of the subjects, the share of energy from proteins, carbohydrates, and total fat was taken into account, in addition to the age, sex, body weight, and Physical Activity Level (PAL) of the subjects.

The standard for energy (EER) was estimated for each subject from the mean value calculated from the results of 3 prediction equations: equation developed by the Institute of Medicine for the assessment of the nutritional status of the subjects based on their sex and age [26]; Mifflin St Jeor equation [27]; and Schofield [28]. Such procedure was performed to avoid higher differences when using particular equations in the case of some participants.

In the calculations made using Mifflin St Jeor and Schofield equations, the total energy demand was estimated by applying PAL in the Food and Agriculture Organization (FAO)/WHO/United Nations University (UNU) interpretation: PAL = 1.4 for sedentary individuals (seated work; lack of physical activity), PAL = 1.6 for individuals with medium physical activity (sedentary work; low physical activity related to standing or walking), PAL = 1.75 for active individuals (work predominantly related to walking, standing, or sitting combined with regular physical activity for about 1 h a day at moderate intensity; active or moderately active lifestyle), and PAL = 2.0 for very active individuals (regular, hard physical work or forced activities during free time over 1 h per day; vigorous or vigorously active lifestyle) [29].

For people below 18 years of age with overweight or obesity, the reference standard was energy intake reduced by300 kcal, and for people >18 years of age with overweight or obesity, an energy deficit of 600 kcal was used [30,31].

### 2.3. Statistical Procedure and Analysis

Statistical analysis was performed using Statistica v13.3 (StatSoft, Kraków, Poland). Due to the heterogeneity of the study group, which includes respondents of various ages, the nutritional status and diet quality were analyzed in the form of a multiple-case study. For each examined person, the implementation of standards for the consumption of dietary components was assessed separately, considering the appropriate standards for a given age group. Data expressed on a quantitative scale were presented as mean with standard deviation (SD). Depending on the result of the Shapiro-Wilk test (assessment of compliance with the normal distribution), the *t*-test, and Mann–Whitney test were used. The size of the effect is given as Cohen’s d factor. Data expressed on a qualitative scale were presented as the number and percentage of the sample. Intergroup differences were assessed using Chi-square (*χ*^2^) test or Fisher exact test. Significance level was established at *p* ≤ 0.05 for the analyses.

## 3. Results

Comparison of BMI determined for the entire study group with the Polish standard from OLA/OLAF programs (including subjects up to 18 years of age) [18] and the WHO classification (for people over 18 years of age) [19] showed that 33.3% of respondents (n = 6) had correct body weight, while abnormally high body weight was found in 66.7% (n = 12), including overweight in 27.8% (n = 5) and obesity in 38.9% (n = 7).

Due to the basic diagnosis of DS (subjects with DS formed a subgroup within the category of subjects with ID), in 11 subjects, the nutritional status was additionally assessed using the BMI standard dedicated to people with DS aged 2–20 years as described by Zemel et al. [19]. This revealed that one person in this group was underweight, three subjects had normal bodyweight, six were overweight, and one was obese. The 12th person with DS did not qualify for the assessment using the standard applied as the person’s age exceeded the applied limits (22 years) and subsequently, the nutritional status was assessed using the WHO classification [32]. Data on the nutritional status of the subjects as well as the information on the basic diagnosis, ID severity, and comorbidities are summarized in Table 1. Statistical analysis with the *χ*^2^ test revealed no statistically significant differences in the frequency of abnormally high body weight in the sex groups (*χ*^2^ = 0.029; *p* = 0.986). Furthermore, no association of the severity of ID with abnormally high body weight was found (*χ*^2^ = 0.34; *p* = 0.56; the analysis included people diagnosed with grade ID, n = 14). The diagnosis of the basic cause of ID (DS (n = 12) versus other diagnoses (n = 6)) also did not differentiate between the subjects in terms of the incidence of abnormally high body weight (*χ*^2^ = 0.28; *p* = 0.6).

Table 2 shows a comparison of the nutritional status diagnosed in each subject with ID with the total energy requirement calculated based on the Institute of Medicine, Mifflin St Jeor, and Schofield equations, considering energy reduction in the case of overweight or obesity (under 18 years of age—300 kcal; over 18 years of age—600 kcal) in relation to the basic diagnosis and degree of ID.

Table 3 summarizes the assessment of energy intake from dietary records in relation to the EER.

Table 4 summarizes the assessment of macronutrient intake from dietary records in relation to the TEE calculated based on the appropriate body mass of the subjects, in whom abnormal body mass was found, i.e., overweight/obesity (n = 11) or underweight (n = 1).

The analysis of vitamin intake with DFR (Table 5) and coverage of vitamin requirements (compliance with the standard) revealed that the intake of vitamins B1, B2, and B6 was excessive in all respondents and exceeded 110% of the EAR. Vitamin C intake corresponded to the EAR norm in one case, while excessive intake was observed in the remaining subjects, with the greatest excess of the norm up to 706.5% of EAR. Regarding the intake of folic acid, four subjects showed a deficient intake, one showed normal intake, and 13 people showed excessive intake. Vitamin B12 intake was close to the norm (90–110% of the norm) and was 105% in only one case, while among 16 subjects (88.9%) excessive intake was found, reaching even 340 and 740% of the norm and in one case a deficient intake at the level of 66.7% of EAR was found. For vitamin A, three persons (16.7%) were characterized by very low intake at the level below 90% of the EAR (37%, 55.9%, and 83.4% of EAR), and in the case of other persons, excessive intake was found (above 110% of the EAR). Vitamin D intake did not meet the Polish standard at the AI level in any of the examined persons with ID. Vitamin E intake was found to be too low in relation to the AI standard among 44.4% of respondents (n = 8).

The results of the dietary intake analysis of various minerals are presented in Table 6. In the vast majority of subjects with ID (n = 17), sodium intake with DFR was sufficient. In 14 subjects (77.8%), the average daily potassium intake reached the AI level with respect to age and gender, while in four, the intake was insufficient. Calcium intake was too low in relation to the EAR norm in as many as 12 subjects (66.7%). Only in two subjects, the intake of calcium was within the normal range (90–110% EAR), while in four, it exceeded 110% of the EAR. On the other hand, phosphorus intake was normal in 2 people (90–110% EAR), while in 16 cases excessive intake (88.9%) was found, exceeding 110% of EAR, and in 3 the intake exceeded 300% of EAR. The intake of magnesium at the EAR level was insufficient in four subjects (22.2%). No deficiencies regarding iron intake were found—in one person, the intake corresponded to the EAR norm (90–110%)—while in 17 people a very high intake exceeding 110% of the EAR value was observed. Zinc intake was within the range of 90–110% of the EAR in three cases, while in the remaining 15 cases it was excessive. Copper intake was in the range of 90–110% of the EAR in only one case, while in the remaining subjects it exceeded 110% of the EAR standard. On the other hand, manganese intake was sufficient among all subjects. Iodine intake differed in the studied group with ID: intake was appropriate only in one person (90–110% of EAR) while in one the intake slightly exceeded this range and the rest showed a deficient intake at low levels, not exceeding 40% of the EAR (in seven people).

The next stage of the analysis evaluated whether there were statistically significant differences in energy and dietary component consumption with respect to gender, as well as baseline diagnosis, ID severity, and nutritional status. The dietary components for which the standard of intake is expressed as range of values (carbohydrates and fats) were excluded from the analysis.

The analysis of differences with respect to gender revealed no statistically significant differences in energy and dietary intake. The basic diagnosis (DS versus other ID causes) also did not differentiate between the respondents. However, significant differences were found in the examined persons based on the severity of ID (the analysis included those subjects for whom the level of ID was determined—n = 14—the analysis did not include carbohydrate and fat intake, for which the reference standard was the range of values—data were presented in Table 7). People with ID of the highest severity (grade 3) showed a significantly lower percentage of EER implementation compared to those with milder severity ID (grades 1 and 2) (*p* = 0.02). In the group with deeper ID, realization of the EAR norm for vitamin B6 was significantly lower (*p* = 0.02). Potassium intake was lower in the group with more severe ID (*p* = 0.006). Similar differences were observed with regard to the intake of phosphorus (*p* = 0.004), iron (*p* = 0.01), copper (*p* = 0.03), iodine (*p* = 0.03), magnesium (*p* = 0.011), and zinc (*p* = 0.046). No differences were noted in the intake of other dietary components depending on the severity of ID. The study also analyzed whether there were significant differences in the intake of nutrients in the groups distinguished by nutritional status (correct nutritional status—n = 6—versus overweight or obesity—n = 12). Vitamin B12 intake was found to be significantly lower in the ID group with abnormally high body weight, compared to the ID group with normal nutritional status (*t*-test; *p* = 0.04). On the other hand, protein intake was higher in the overweight or obese group than those with normal body weight (*U*-test; rank-sum *p* = 0.04). No statistically significant differences were observed in the intake of other dietary components.

## 4. Discussion

Due to the lack of Polish data regarding the diet quality of children, adolescents, and young adults with ID, considering the intake of various dietary components, it is impossible to confront the results of this study with the findings of other Polish authors. The discussion referred mainly to foreign studies in which the research group and applied methodology were similar [3,5,33].

The results of this study showed that excess body weight was observed in 66.7% and obesity in 38.9% of the respondents (seven subjects). Other Polish study also indicated high prevalence of obesity among people with ID [9]. This is comparable to the results of the present research conducted among the younger population. According to the Centers for Disease Control and Prevention (CDC) data, the rate of obesity among adults with disabilities is 36%, and among people without disabilities it is 23%. In the group aged 2–17, the rates were found to be as follows: children with disabilities: 22% and children without disabilities: 16% [34]. Marks [10] estimated that children with DS constitute 5–6% of the total number of obese children. Mikulovic et al. [35] estimated the prevalence of excess body weight in a French population of children and adolescents with ID at 19%, and noted that the percentage was higher in the 15- to 20-year-old group of older adolescents. In a Polish study conducted by Podgórska-Bednarz et al. [36], the prevalence of excess body weight among children and adolescents with ID (7–18 years old) was estimated at 31.6%, while in the control group of children without ID the prevalence was 20.7%. The risk of developing excess body weight in the ID group was almost two-fold higher (odds ratio = 1.77) than in healthy children and increased with the severity of ID. In turn, in an Australian study, De et al. [37] observed overweight in 40% of children with developmental disabilities, and obesity in 15%. The study of Segal et al. [38] on an American population indicated that the prevalence of obesity among youth with ID was almost double compared to the general population. The above data highlight a high risk of excess body weight in people with ID [39].

The present study did not find any differences in the occurrence of excess body weight depending on gender, basic diagnosis, or the severity of ID. Similarly, in the studies by Gawlik et al. [9] and De et al. [37], no correlation was noticed between the nutritional status and the grade of ID. In the study of Hoey et al. [1], waist circumference was observed to increase with an increase in the degree of impairment, but no such correlation was found with BMI. On the other hand, a Norwegian study [40] showed that deeper impairment is associated with malnutrition, while milder impairment is associated with excess body weight, which would result from food refusal and self-induced vomiting in the case of more severely disabled people.

Regarding the intake of macronutrients with the diet the intake of proteins, total fats, and carbohydrates was found to be normal in most of the studied cases, while the intake of SFA was found to be excessive. The results on dietary macronutrient intake vary significantly in the available studies on ID populations. For example, a Brazilian study by Magenis et al. [3] showed that among people with ID (mean age 9.94 ± 4.28 years), protein intake was excessive in 94.7%, carbohydrate intake in 78.9%, and fat intake in 84.2%, with excess protein and fat intake being more frequent in the ID group than in the control group (people without ID). In a Saudi study performed by Abdallah et al. [33], the intake of energy, proteins, carbohydrates, and fats was significantly higher in the group of children with DS compared to the control group. A study by Grammatikopoulou et al. [5] in a Greek population of children and adolescents with ID revealed excessive protein intake and very high energy intake from carbohydrates, contributing to 57–60% of energy. The study showed that the Greek ID subjects received high-carbohydrate and low-fat diets. In the Irish SOPHIE study by Hoey et al. [1], conducted in a group of 131 people with ID (16–64 years), a high prevalence of excess body weight was found (28.2% were overweight and 46.8% were obese), as well as higher energy consumption from sugars and fats, including SFA. On the other hand, a Spanish study by Soler Marin et al. [41] conducted in a group of young adults with DS (n = 38) indicated that energy coverage was 16.3 and 18.8% from protein, 38.1 and 37.9% from fat, and 45.6 and 43.3% from carbohydrates among men and women, respectively. The study group showed high prevalence of overweight (36.8%) and obesity (36.8%).

The results of the present study on excessive SFA intake by the respondents with ID should be interpreted in the context of the eating habits of the Polish population, which include a relatively high amount of animal fats (56% of men and 30% of women consume meat products with visible fat) [42], resulting in a high intake of SFA. The WOBASZ II study showed that SFA covered 13.8% of the daily energy requirement in adult men (SD ± 4.3) and 13.3% in women (SD ± 4.4) [42], which is higher than the recommended standard.

The present study showed that the intake of fiber in most of the respondents corresponded to the AI standard. Contrastingly, the study performed by Grammatikopoulou et al. [5] in the Greek population of children and adolescents with ID showed insufficient fiber intake among the subjects. In turn, in the Saudi research of Abdallah et al. [33] in the group of people with ID, the intake of fiber was significantly higher than in the control group, whereas the Brazilian study by Magenis et al. [3] showed that most of the studied people with DS reported fiber intake below recommendations (AI). In the study of Hoey et al. [1] conducted on a group of 131 people with ID (aged 16–64 years), the mean intake of fiber was lower than recommended.

The results of the present research revealed excessive intake of B vitamins (B1, B2, B3, B6, B12) in all or the vast majority of respondents. On the contrary, Mazurek et al. [16] revealed deficiencies in the intake of B vitamins in people with DS. A similar situation of excessive intake was found for vitamin C in the vast majority of studied people, while deficient folic acid intake was found only in 4/18 and vitamin A in only 3/18. Assuming that dietary records may lead to the underestimation of intake compared to other questionnaire methods [2], actual vitamin intake may be even higher than reported. The study of Grammatikopoulou et al. [5] showed that the intake of B vitamins, in both Greek children and adolescents with ID, exceeded the RDA (for vitamins B1, B2, B3, B6, and B12, the intake was 177.3, 294.6, 132.3, 241.4% and 345.7% of the RDA, respectively, for children with ID and 165.17, 206.1, 109.8, 143.9, and 198% of the RDA for adolescents with ID). This result corresponds to those of the present study. The study of Grammatikopoulou et al. also evaluated vitamin C intake (reference to the RDA standard) [5]: intake was above the recommended level in both groups with ID (383.8% of the RDA in children, 226.4% of the RDA in adolescents). The intake of folic acid in the cited Greek study was deficient in both groups (81.1 and 84.4% of the RDA, respectively), while in the present study, only four subjects were characterized by intake lower than 90% of the EAR. The assessment of vitamin A intake showed similar results in the Polish and Greek populations with ID, indicating generally sufficient intake (in the present research, deficient intake was observed only in three people). Similar to the present study, Grammatikopoulou et al. [5] revealed insufficient intake of vitamin D with the diet (70.1% of the RDA in children, 94.3% of the RDA in adolescents). In the present study, all the subjects showed a deficient intake of this vitamin in relation to the AI standard. On the other hand, the findings of Greek researchers [5] regarding “alarmingly low vitamin E consumption” were not supported by the present study. The Brazilian research by Magenis et al. [3] revealed the highest percentage of people with a deficient intake of vitamin B5 (pantothenic acid) (not assessed in the present study), as well as the following vitamins: folic acid and vitamin B3. Similar to the present study, the Brazilian study did not reveal vitamin C deficiency, but confirmed the high prevalence of insufficient vitamin D intake [3]. Regarding the intake of vitamins in people with ID, the Saudi research of Abdallah et al. [33] assessed only the intake of vitamins A and C. The intake of vitamin A in most of the subjects with ID (63.4%) was at a level below 75% of the RDA standard, while the intake of vitamin C in the vast majority of respondents (96.6%) was adequate or excessive.

The analysis of the intake of dietary minerals revealed the highest deficiencies in the intake of calcium and iodine. The remaining minerals were consumed in excess amounts by most of the respondents. Deficient calcium, iron, potassium and zinc intake was revealed in the Italian study by Bertoli et al. [2]. The nutrients that were reported by Bertoli et al. as deficient in their study were consumed in excessive amounts (iron, zinc) by the subjects in the present study. The consumption of potassium in the present study was sufficient in the majority of the respondents. Similar to the present study, the Greek research by Grammatikopoulou et al. [5] revealed deficient iodine levels, but only in the diet of adolescents with ID, as well as deficient calcium—also in this group. This result may suggest the effectiveness of parental supervision over the nutrition of children with ID, but not of adolescents with ID. Study by Abdallah et al. [33] revealed a deficient intake of calcium and zinc. In turn, the iron intake was below 75% of the RDA in 50% of the respondents, while in 30% it was 100% of the RDA and in 20% it exceeded 100% of the RDA. On the other hand, the study by Magenis et al. [3] showed that children and adolescents with DS had excessive sodium intake and deficient calcium intake, while none of the subjects showed deficient iron intake and only one showed deficient zinc intake. In the study of Hoey et al. [1], conducted among people with ID, deficiency of calcium intake was demonstrated in the majority of respondents.

In the present study, deficient calcium intake observed in most of the respondents (12/18) should be interpreted in the context of increase risk of developing osteoporosis among people with ID. With a chronic deficient intake of calcium, its level in the circulating blood is maintained, among others, by bone resorption, which may lead to bone loss and osteopenia/osteoporosis [2]. This process is additionally intensified in this population by low level of physical activity. However, in people with DS (trisomy 21), the mechanism of enhanced calcium absorption via the passive route may act to protect against the effects of deficient calcium intake [43].

When interpreting the results obtained for deficiencies in the intake of dietary micronutrients, one should pay attention to their relationship with cognitive function. Many scientific data have suggested an important role for folic acid, as well as other metabolically related B vitamins (B2, B6, B12), in delaying the progress of cognitive decline in brain aging processes [44,45]. The analysis of the intake of the above-mentioned vitamins in the studied sample showed that most of the respondents met the norm. The studied group did not show any deficiencies in iron intake that could lead to iron deficiency anemia, associated with motor and cognitive developmental deficits in children, especially those with ID [46].

On the other hand, zinc intake should be considered in the context of metabolism disorders of this element in people with DS. In contrast to results of the present study, a research by Lima et al. [47] on 35 children with DS showed adequate intake of this element only in 40% of children. The results of the present study indicated that there was no person with inadequate zinc intake in the studied population.

An important point of reference for the results of the present study is those obtained in the population of healthy children in Poland. The assessment of food rations of healthy Polish children aged 10–12 showed insufficient intake of proteins and carbohydrates, and excessive intake of fats (including SFA) and sucrose. The intake of dietary fiber and calcium was also very low, which accounted for 50% of the average demand in the case of both nutrients [48,49,50,51,52].

Other available Polish studies are not suitable for comparison with the results of the present research, due to their different methodology, which was based on the use of author-designed, non-standardized questionnaires, which evaluated rather the dietary habits and the frequency of consumption of the specific groups of products, than actual food intake [53,54].

Referring to the presented results indicating statistically significant differences in the implementation of the norm of intake of various nutrients in groups differing in the intensity of ID (lower intake of certain diet components in the group with deeper ID), it could be concluded that, to the best of our knowledge, this study is the first to evaluate the intake of various nutrients in terms of ID severity. The studies, cited in this paper, whose profile is similar to the present research, do not contain data in this regard [1,2,3,5,33]. There is only information on the prevalence and specificity of eating/feeding disorders among people with various levels of ID, for example, in the Israeli research conducted by Gal et al. [55]. This study revealed that significant differences were noted in aspiration risk (highest risk in the severe mental retardation group) and feeding/eating functions (severity increased with that of ID). However, no studies comparing the intake of different nutrients in relation to the severity of ID can be found in the scientific literature.

The present study has certain limitations. The main limitation is the small size of the study group, resulting from difficult access to people with such conditions, as well as the wide age range of participants. However, most studies with a similar profile also included small research groups: Magenis et al. [3]—n = 19; Abdallah et al. [33]—n = 30; Grammatikopoulou et al. [5]—n = 30; and Hamzaid et al. [56]—n = 33. The wide age distribution of the respondents was partially solved by referring each time to the age-specific consumption norms.

It should be also remembered that the intake of micronutrients based on the 3-days dietary survey is not fully reliable, and this is also a limitation of the present study.

However, these are preliminary studies, which, based on the obtained results, might be very interesting to the scientific community. The strength of the present study is that it compared the dietary intake of nutrients with the anthropometric and clinical status of the respondents, taking into account the nutritional status assessed based on the measured anthropometric parameters, and not based on declaration by the respondents or their guardians. It must be mentioned that parents/guardians of all people examined in this study received individual dietary advice, aimed at the correction of any irregularities in the diet.

## 5. Conclusions

Results of the present study revealed that compared to people with less severe ID (grades 1 and 2), people with grade 3 ID showed significantly lower intake of dietary components such as vitamin B6, potassium, phosphorus, iron, copper, iodine, magnesium, and zinc. However, the intake of phosphorus was excessive in most studied ID individuals, and the intake of vitamin B6, iron, copper, iodine, magnesium and zinc was excessive in all studied ID individuals.

No significant differences were noted in the nutritional reference fulfillment for various nutrients among the groups distinguished by sex, nutritional status, or the cause of ID.

Further studies are recommended on energy intake and dietary components in the population with ID in Poland, taking into account groups of persons with ID selected because of the same underlying disease.

## Figures and Tables

**Table 1:** Characteristics of the studied participants.

Category	Distribution	n	%
**Gender**	Male	11	61.1
	Female	7	38.9
**Level of intellectual disability**	Mild	1	5.6
	Moderate	4	22.2
	Severe	9	50.0
	No data ^1^	4	22.2
**Diagnosis**	Down syndrome (DS)	12	66.7
	Autism spectrum disorder (ASD)	3	16.7
	Cerebral palsy (CP)	1	5.6
	Kleefstra syndrome (KS)	1	5.6
	Genetic disorder undefined (GD)	1	5.6
**Assessment of the nutritional status of people with Down syndrome using the American standard (percentile grids for BMI) ^2^ [20]**	Underweight	1	5.6
	Normal weight	3	16.7
	Overweight	6	33.3
	Obesity	1	5.6
	Not applicable ^3^	7	38.9
**Assessment of nutritional status using the Polish standard (percentile grids for BMI) ^4^**	Underweight	0	0.0
	Normal weight	6	33.3
	Overweight	2	11.1
	Obesity	7	38.9
	Not applicable ^5^	3	16.7
**Comorbidities**	Lack	2	11.1
	Hypothyroidism	11	61.1
	Heart defects	3	16.7
	Cholecystolithiasis	3	16.7
	Defect of vision	4	22.2
	Psoriasis	1	5.6
	Allergy	3	16.7
	Epilepsy	3	16.7
	Kidney stones	1	5.6
	Neurogenic bladder	1	5.6
	Other	7	38.9

^1^ The level of intellectual disability in younger children has not yet been determined at the time of data collection ^2^ The assessment concerned respondents with DS, aged 2–20 years (n = 11). ^3^ This category includes people with ID who do not have a diagnosis of DS, as well as people with DS over the age of 20. ^4^ Polish standards were used to assess the nutritional status of the subjects with ID, aged 3–18 (n = 15). ^5^ This category included participants over 18 years of age. Their nutritional status was as follows: person number 3: overweight (BMI 27.6); person 7: 1st degree obesity (BMI 32.1); person 8: 2nd degree obesity (BMI 35.4). Statistical analysis was performed using Chi-square (*χ*^2^) test at *p* ≤ 0.05.

**Table 2:** Nutritional status in the studied group in relation to the total energy requirement.

No	Age	Basic Diagnosis	ID Severity	Body Mass (kg)	Height (m)	BMI (kg/m^2^)	IOM	Schofield’s Equation	Mifflin’a St Jeors Equation	Nutritional Status [20]	Nutritional Status (OLA/OLAF, BMI)
1	17	DS	3	50	1.55	20.8	2151.35	2468.0	2222	norm	norm
2	11	DS	3	59.7	1.45	28.4	1848.62	1788.3	2059.6	overweight	obesity
3	22	DS	3	59.7	1.47	27.6	2293.21	1579.7	1975.05	n.a.	overweight
4	17	DS	3	45.4	1.47	21.0	1756	1820.3	1802.8	norm	norm
5	14	DS	3	73.9	1.53	31.6	2619.39	2451.3	2608.4	overweight	obesity
6	16	DS	3	62.2	1.47	28.8	1694.15	1835.1	2079.6	norm	overweight
7	22	DS	3	70.3	1.48	32.1	2442.59	2201.1	2132.2	n.a.	obesity
8	19	DS	3	76.5	1.47	35.4	2078.98	1668.2	1713.3	overweight	obesity
9	4	DS	1	17.4	0.936	19.9	1141.9	1043.0	924.8	overweight	overweight
10	4	DS	2	12.3	0.92	14.5	1196.19	1177.2	827.2	underweight	norm
11	8	DS	2	35	1.28	21.4	1482.45	1615.1	1518.4	overweight	overweight
12	17	CP	2	73	1.53	31.2	2449.14	2429.0	2570	n.a.	obesity
13	17	DS	3	83.3	1.57	33.8	2696.45	2684.0	2774.8	overweight	obesity
14	12	ASD	No data	42.5	1.52	18.4	2203.96	2255.8	2112	n.a.	norm
15	7	ASD	No data	35.6	1.29	21.4	2213.19	1800.2	1811.6	n.a.	overweight
16	5	ASD	No data	20.5	1.17	15.0	1407.86	2260.5	1466	n.a.	norm
17	12	KS	No data	48.9	1.55	20.4	2075.98	2132.3	2244.4	n.a.	norm
18	9	GD	2	63	1.405	31.9	2516.17	2408.7	2349	n.a.	obesity

Data were calculated according to the Institute of Medicine, Mifflin-St Jeor’s and Schofield’s equations, taking into account the energy deficit in the case of indications, i.e., presence of overweight or obesity (under 18 years of age—300 kcal, over 18 years of age—600 kcal), basic diagnosis and the degree of intellectual disability. Abbreviations: Down Syndrome (DS), Autism spectrum disorder (ASD), Kleefstra syndrome (KS), Cerebral palsy (CP), Genetic disorder undefined (GD), n.a.—not applicable.

**Table 3:** Assessment of energy and macronutrient intake in people with ID.

	**Energy (kcal)**			**Protein (g)**					**Fats (g)**		
**No**	**Mean Daily Intake**	**EER**	**Compliance with the Standard (%)**	**Mean Daily Intake** **(g)**	**10–20% of EER (g)**	**Compliance with the Standard**	**Estimated Average Requirement (EAR) (g/person/day)**	**Compliance with the Standard (%)**	**Mean Daily Intake**	**20–35% of EER (g)**	**Compliance with the Standard**
1	1761.8	2280.5	77.3	78.8	57–114	Norm	40.5	194.6	70	50.7–88.7	Norm
2	2079.1	1898.8	109.5	119.4	47.5–94.9	Above	50.1	238.3	78.4	42.2–73.8	Norm
3	1872.2	1949.3	96	71.3	48.7–97.5	Norm	43.6	163.5	70	43.3–75.8	Norm
4	1958.4	1793	109.2	76.5	44.8–89.7	Norm	35.9	213.1	63.5	39.8–69.7	Norm
5	2700.5	2559.7	105.5	158	64–128	Above	62.1	254.4	75	56.9–99.5	Norm
6	1926.8	1869.6	103.1	71.6	46.7–93.5	Norm	49.1	145.8	74.7	41.5–72.7	Norm
7	2193.6	2258.6	97.1	95.5	56.5–112.9	Norm	51.3	186.2	98.2	50.2–87.8	Above
8	1198.3	1820.2	65.8	62	45.5–91	Norm	55.8	111.1	40	40.4–70.8	Norm
9	1741.4	1036.6	168	53	25.9–518	Norm	14.6	363	48.6	23–40.3	Above
10	1883.6	1066.9	176.6	69	26.7–53.3	Above	10.3	669.9	46.2	23.7–41.5	Above
11	1487.8	1538.6	96.7	70.7	38.5–76.9	Norm	29.4	240.5	37.5	34.2–59.8	Norm
12	3095.3	2482.7	124.7	97.2	62.1–124.1	Norm	59.1	164.5	120.6	55.2–96.5	Above
13	1811.3	2718.4	66.6	76.9	67.9–135.9	Norm	67.5	113.9	46	60.4–105.7	Above
14	3137.1	2190.6	143.2	119	54.8–109.5	Norm	35.7	333.3	129.7	48.7–85.2	Above
15	1108.1	1941.7	57.1	48.6	48.5–97.1	Norm	29.9	162.5	22.4	43.1–75.5	Above
16	2408.1	1711.4	140.7	125.3	42.8–85.6	Above	17.2	728.5	73.7	38–66.6	Above
17	2130	2150.9	99	97.6	53.8–107.5	Norm	41.1	237.5	78.4	47.8–83.6	Norm
18	2333	2424.6	96.2	116.7	60.6–121.2	Norm	52.9	220.6	96.3	53.9–94.3	Norm
**No**	**SFA (g)**			**Carbohydrates (g)**				**Dietary Fiber (g)**			
	**Mean Daily Intake**	**6% of EER (g)**	**Compliance with the Standard (%)**	**Mean Daily Intake**	**Recommended Intake** **45–65% of EER**		**Compliance with the Standard**	**Mean Daily Intake**	**AI (g)**	**Compliance with the Standard (%)**	
1	19.1	15.2	Above	196.5	256.6–370.6		Under	17.7	21	D	
2	24	12.7	Above	212.7	213.6–308.6		Norm	26.5	19	E	
3	31.3	13	Above	228.3	219.3–316.8		Norm	20.2	25	D	
4	23.6	12	Above	262.6	201.7–291.4		Norm	15.5	21	D	
5	23.7	17.1	Above	322.6	288–416		Norm	55.1	19	E	
6	24.8	12.5	Above	230.2	210.3–303.8		Norm	27.8	21	E	
7	37.3	15.1	Above	223.1	254.1–367.0		Under	19.7	25	D	
8	18	12.1	Above	142.2	204.8–295.8		Under	13.4	25	D	
9	12	6.9	Above	262.8	116.6–168.4		Above	22.9	14	E	
10	16.7	7.1	Above	199.7	120–173.4		Above	17.7	14	E	
11	10.9	10.3	Norm	205.6	173.1–250		Norm	25	16	E	
12	54.1	16.6	Above	393.5	279.3–403.4		Norm	25.9	21	E	
13	15.8	18.1	Norm	256.7	305.8–441.7		Under	38.3	21	E	
14	41.3	14.6	Above	356.4	246.4–356		Norm	34.1	19	E	
15	7.7	12.9	Norm	173	218.4–315.5		Under	30	16	E	
16	24.7	11.4	Above	298.8	192.5–278.1		Norm	30.7	14	E	
17	24.7	16.3	Above	244.8	242–349.5		Norm	26.5	19	E	
18	31.2	16.2	Above	237.3	272.8–394		Under	21.9	16	E	

**Table 4:** Assessment of macronutrient intake in subjects with ID with abnormal body mass with reference to Total Energy Requirement (TEE) calculated for appropriate body mass.

	Energy	Protein			Fat			Carbohydrates		
No ^1^	TEE ^2^ (kcal)	Protein Mean Daily Intake (g)	10–20% of TEE (g) ^3^	Compliance with the Standard	Mean Daily Intake	20–35% of TEE (g) ^3^	Compliance with the Standard	Mean Daily Intake	Recommended Intake 45–65% of TEE ^3^	Compliance with the Standard
2	2206.5	119.4	55.2–110.3	above	78.4	49.0–85.8	norm	212.7	248.2–358.6	under
3	2954.5	71.3	73.9–147.7	under	70	65.7–114.9	norm	228.3	332.4–480.1	under
5	2954.5	158	73.9–147.7	above	75	65.7–114.9	norm	322.6	332.4–480.1	norm
7	2978.9	95.5	74.5–148.9	norm	98.2	66.2–115.8	norm	223.1	335.1–484.1	under
8	2588.5	62	64.7–129.4	under	40	57.5–100.7	under	142.2	291.2–420.6	under
9	1135.6	53	28.4–56.8	norm	48.6	25.2–44.2	above	262.8	127.8–184.5	above
10	1008.7	69	25.2–50.4	above	46.2	22.4–39.2	above	199.7	113.5–163.9	above
11	1794.7	70.7	44.9–89.7	norm	37.5	39.9–69.8	under	205.6	201.9–291.6	norm
12	2786.4	97.2	69.7–139.3	norm	120.6	61.9–108.4	above	393.5	313.5–452.8	norm
13	3211.5	76.9	80.3–160.6	under	46	71.4–124.9	under	256.7	361.3–521.9	under
15	1832.5	48.6	45.8–91.6	norm	22.4	40.7–71.3	under	173	206.2–297.8	under
18	2084.5	116.7	52.1–104.2	above	96.3	46.3–81.1	above	237.3	234.5–338.7	norm

^1^ The Table took into account the subjects with ID with improper body mass. ^2^ TEE was estimated from the equations provided in the norms of nutrition for Polish population (Males = 310.2 + 63.3 × W − 0.263 × W^2^; Females = 263.4 + 65.3 × W − 0.454 × W^2^) based on appropriate body mass. ^3^ Macronutrient intake was related to the protein norm expressed as the gram equivalent of TEE percentage and assessed as normal, excessive or deficient.

**Table 5:** Dietary intake of selected vitamins with DFRs among people with ID.

**No**	**Vitamin B1 (EAR)**		**Vitamin B2 (EAR)**		**Vitamin B3 (EAR)**			**Vitamin B6 (EAR)**			**Folic Acid (EAR)**		
	**Mean Intake** **(mg)**	**Compliance with the Standard (% EAR)**	**Mean Intake** **(mg)**	**Compliance with the Standard (% EAR)**	**Mean Intake** **(mg)**		**Compliance with the Standard (% EAR)**	**Mean Intake** **(mg)**		**Compliance with the Standard (% EAR)**	**Mean Intake (µg)**		**Compliance with the Standard (% EAR)**
1	1.7	170.0	1.3	118.2	17		141.7	1.7		154.5	272.7		82.6
2	1.7	212.5	3	375.0	20.6		228.9	2.7		270.0	469.7		187.9
3	1.5	136.4	1.6	145.5	18.2		151.7	2.6		236.4	377.5		118.0
4	1.3	144.4	1.5	166.7	11.9		108.2	1.5		150.0	213.3		64.6
5	3.4	340.0	3	272.7	42.5		354.2	4.2		381.8	666.9		202.1
6	2.3	255.6	1.6	177.8	18.6		169.1	2.2		220.0	490.9		148.8
7	2.1	190.9	1.7	154.5	22.3		185.8	2.7		245.5	366.3		114.5
8	1.3	144.4	1.1	122.2	14.1		128.2	1.6		145.5	225.1		70.3
9	1.5	300.0	1.6	320.0	20.8		346.7	2.3		460.0	351.1		219.4
10	1.2	240.0	1.8	360.0	18.5		308.3	2.2		440.0	255		159.4
11	1	142.9	1.9	237.5	17.2		191.1	2.4		300.0	402.7		161.1
12	2.3	230.0	2.2	200.0	22.1		184.2	2.5		227.3	411.4		124.7
13	1.6	160.0	1.6	145.5	18.1		150.8	2.1		190.9	381.9		115.7
14	2.2	244.4	2.3	255.6	27.5		305.6	2.5		250.0	477.5		191.0
15	1.2	171.4	0.9	112.5	18.7		207.8	1.7		212.5	209.04		83.6
16	2.5	500.0	2.4	480.0	30.3	6	505.0	3.4	0.5	680.0	411.1	160	256.9
17	1.9	211.1	1.8	200.0	20.4	9	226.7	2.4	1	240.0	257.3	250	102.9
18	2.2	314.3	2.1	262.5	23.5	9	261.1	2.5	0.8	312.5	309.9	250	124.0
**No**	**Vitamin B12 (EAR)**		**Vitamin C (EAR)**		**Vitamin A (Retinol Equivalent, EAR)**			**Vitamin D (AI)**			**Vitamin E (AI)**		
	**Mean Intake (µg)**	**Compliance with the Standard (% EAR)**	**Mean Intake (mg)**	**Compliance with the Standard (% EAR)**	**Mean Intake (µg)**	**Compliance with the Standard (% EAR)**		**Mean Intake (µg)**	**Compliance with the Standard**		**Mean Intake (mg)**	**Compliance with the Standard**	
1	2.9	145.0	135.7	208.8	909.8	144.4		1.4	D		9.6	D	
2	4.6	306.7	140.8	352.0	935.7	217.6		6	D		11.1	E	
3	2.5	125.0	265.5	354.0	2750.8	436.6		2.1	D		12.2	E	
4	2.9	145.0	84.6	153.8	802.3	163.7		1.2	D		6.2	D	
5	4.2	210.0	353.1	543.2	2185.1	346.8		1.1	D		16.8	E	
6	2.8	140.0	195.8	356.0	1401	285.9		2.3	D		16.1	E	
7	3.5	175.0	309.5	412.7	1080.9	171.6		1.7	D		9	D	
8	2.4	120.0	71.6	119.3	762.3	152.5		1.8	D		5.5	D	
9	1.3	130.0	176.1	440.3	167.6	55.9		4.7	D		14.1	E	
10	2.9	290.0	69.7	174.3	412.5	137.5		2.2	D		8.4	E	
11	3.6	240.0	282.6	706.5	1228.4	351.0		2.9	D		6.9	D	
12	3.9	195.0	98.9	152.2	855	135.7		4.3	D		13.9	E	
13	2.1	105.0	183.2	281.8	233.1	37.0		1.1	D		7.3	D	
14	5.1	340.0	176.7	441.8	569	126.4		3.7	D		15.9	E	
15	1	66.7	108.6	271.5	825.1	235.7		0.2	D		4.5	D	
16	7.4	740.0	105.4	263.5	579.8	193.3		10.8	D		12.2	E	
17	4.2	280.0	40.1	100.3	845.1	187.8		4.1	D		7.4	D	
18	3.5	233.3	135.8	339.5	292	83.4		1.7	D		11	E	

Abbreviations: E—enough intake; D—deficiency.

**Table 6:** Dietary intake of selected minerals with DFRs among people with ID.

**No**	**Sodium**		**Potassium**		**Calcium**		**Phosphorus**		**Magnesium**	
	**Mean Intake (mg)**	**Compliance with the Standard**	**Mean Intake (mg)**	**Compliance with the Standard**	**Mean Intake (mg)**	**Compliance with the Standard (% EAR)**	**Mean Intake (mg)**	**Compliance with the Standard (% EAR)**	**Mean Intake (mg)**	**Compliance with the Standard (% EAR)**
1	2703.5	E	2687.6	D	561.4	51.0	1021.7	97.3	262.1	77.1
2	2165	E	4726.4	E	1410	128.2	2132.6	203.1	401.3	200.7
3	2998.4	E	3320.2	D	467.8	58.5	993.2	171.2	232.9	70.6
4	1890.9	E	1986.7	D	693.9	63.1	1007.6	96.0	219.6	73.2
5	4358.9	E	6764.7	E	1392.8	126.6	2899.5	276.1	696.6	204.9
6	2592.3	E	3643.6	E	735.7	66.9	1192.1	113.5	297.5	99.2
7	3452.6	E	3937.1	E	836.8	104.6	1338.9	230.8	309.6	93.8
8	1543.5	E	2183.9	D	382.6	47.8	791.5	136.5	170.3	66.8
9	1810.5	E	3576.2	E	642.8	80.4	1033.8	252.1	283	257.3
10	1986.9	E	3057.9	E	661.5	82.7	1330.6	324.5	322.6	293.3
11	2309	E	4081.2	E	678.9	84.9	1161.3	232.3	334.8	304.4
12	3098.8	E	4400.5	E	912.3	82.9	1444.3	137.6	364	107.1
13	1412	D	3531.1	E	650	59.1	1480.9	141.0	463	136.2
14	4173.5	E	3756.4	E	1032.1	93.8	1756.2	167.3	393	196.5
15	1201.7	E	2282.7	E	398.1	49.8	768.5	153.7	224.4	204.0
16	2108.1	E	4592.3	E	898.6	112.3	1853.1	452.0	393.2	357.5
17	3049.6	E	2970.9	E	281.1	25.6	1640.8	156.3	377.5	188.8
18	3225.8	E	3896.3	E	921.8	115.2	1505.8	301.2	327.4	297.6
**No**	**Iron**		**Zinc**		**Copper**		**Iodine**		**Manganese**	
	**Mean Intake (mg)**	**Compliance with the Standard (% EAR)**	**Mean Intake (mg)**	**Compliance with the Standard (% EAR)**	**Mean Intake (mg)**	**Compliance with the Standard (% EAR)**	**Mean Intake [µg]**	**Compliance with the Standard (% EAR)**	**Mean Intake (mg)**	**Compliance with the Standard**
1	13.3	166.3	9.9	116.5	1.1	157.1	20.8	21.9	5.3	E
2	13.2	188.6	13.9	198.6	1.3	260.0	26	34.7	4.1	E
3	11.2	186.7	9.6	102.1	1.1	157.1	34	35.8	4.1	E
4	9	112.5	8.4	115.1	1	142.9	27	28.4	2.4	E
5	22.5	281.3	20.9	245.9	2.4	342.9	58.7	61.8	12.3	E
6	14	175.0	10.3	141.1	1.3	185.7	47.1	49.6	5.2	E
7	12	200.0	10.2	108.5	1.3	185.7	63.6	66.9	3.5	E
8	7.2	90.0	7.2	105.9	0.7	100.0	49.9	52.5	3	E
9	16.1	402.5	8.2	205.0	1.5	500.0	73.2	112.6	3.7	E
10	9.4	235.0	8.3	207.5	1	333.3	50	76.9	2.5	E
11	12.2	305.0	8.9	222.5	1.2	240.0	41	58.6	4.2	E
12	15.5	193.8	11.3	132.9	1.6	228.6	45.6	48.0	5.7	E
13	15.2	190.0	11.9	140.0	1.8	257.1	32.5	34.2	8.4	E
14	17.1	244.3	14.1	201.4	1.8	360.0	54.9	73.2	7.2	E
15	7.2	180.0	5.2	130.0	0.9	180.0	22.7	32.4	4.1	E
16	17	425.0	13.7	342.5	1.6	533.3	70.9	109.1	5.8	E
17	14.3	204.3	14.5	207.1	1.3	260.0	18.8	25.1	7.2	E
18	11	275.0	14.5	362.5	1.4	280.0	39.6	56.6	3.7	E

Abbreviations: E—enough intake, D—deficiency.

**Table 7:** Comparison of the energy and nutrients intake (in terms of the norm realization) in groups distinguished according to the severity of ID (differences statistically significant excluding fats and carbohydrates). Data Shown as mean ± SD.

Parameter (Mean Values)	ID 3 Severity (n = 9)	ID Severity 1 and 2 (n = 5)	*p*	Cohen’s d
Energy (% of EER) *	92.23 ± 17.17	132.44 ± 38.29	0.018	1.52
Vitamin B6 (% of EER) *	221.62 ± 75.13	347.96 ± 98.92	0.019	1.44
Potassium (% of AI) *	114.52 ± 58.38	234.40 ± 74.70	0.006	1.79
Phosphorus (% of EAR) *	162.83 ± 62.75	249.54 ± 72.67	0.037	1.28
Iron (% of EAR) *	176.71 ± 54.37	282.26 ± 79.19	0.012	1.55
Copper (% of EAR) *	198.72 ± 74.68	316.38 ± 110.53	0.034	1.25
Iodine (% of EAR) *	42.87 ± 15.47	70.54 ± 25.76	0.026	1.30
Magnesium (% of EAR sum of rangs) **	113.61 ± 54.81	251.94 ± 82.99	0.011	1.97
Zinc (% of EAR sum of rangs) **	141.52 ± 49.24	226.08 ± 83.81	0.046	1.23

* *t* Student’s test, ** U Mann-Whitney test.

## Data Availability

Dietary records of researched ID persons available on request (in Polish).
